# Morphostructural Characterization of the Black Creole Goat Raised in Central Mexico, a Currently Threatened Zoogenetic Resource

**DOI:** 10.3390/ani9070459

**Published:** 2019-07-19

**Authors:** Juan Carlos Silva-Jarquin, Sergio Iván Román-Ponce, Marina Durán-Aguilar, Héctor Raymundo Vera-Ávila, Víctor Hugo Cambrón-Sandoval, Héctor Mario Andrade-Montemayor

**Affiliations:** 1 Doctorado en Ciencias Biológicas, Facultad de Ciencias Naturales, Universidad Autónoma de Querétaro. Avenida de las Ciencias S/N Juriquilla, Delegación Santa Rosa Jáuregui, Querétaro, C.P. 76230, México; 2 Centro Nacional de Investigación Disciplinaria en Fisiología y Mejoramiento Animal. INIFAP—SADER. Km. 1 Carretera a Colón, Ajuchitlán, Querétaro, C.P. 76280, México; 3 Licenciatura en Medicina Veterinaria y Zootecnia, Facultad de Ciencias Naturales, Universidad Autónoma de Querétaro. Avenida de las Ciencias S/N Juriquilla, Delegación Santa Rosa Jáuregui, Querétaro, C.P. 76230, México; 4 Licenciatura en Horticultura Ambiental, Facultad de Ciencias Naturales, Universidad Autónoma de Querétaro. Avenida de las Ciencias S/N Juriquilla, Delegación Santa Rosa Jáuregui, Querétaro, C.P. 76230, México

**Keywords:** Creole goat, morphology, zoometry, characterization

## Abstract

**Simple Summary:**

The need to characterize and document local animal populations has gradually gained global importance. This is because these populations represent a genetic pool, and before developing strategies for their conservation, they need to be evaluated. The Black Creole goat, which is distributed mainly in the central region of Mexico, represents one of the first Creole populations, derived from goat cattle introduced 500 years ago by Spanish colonizers. However, morphological and racial standards have not been established for this population, even though the quality of their milk and the social importance they represent in semi-desert areas are known. In the present study, the morphostructure of the Black Creole goat was evaluated using morphometric variables that describe the body conformation of the animals. The results showed a homogeneous population and confirmed the zootechnical purpose of the animals. These results represent the first morphological study carried out on the Black Creole goat and could be the basis for establishing its racial standard.

**Abstract:**

In order to evaluate the morphostructural variability of the Black Creole goat (BCG), the present study was carried out in a population of 226 animals from eight localities and 14 morphometric variables were taken. Descriptive statistics for the variables were obtained and 10 of these presented variation coefficients of less than 10%. The degree of harmony in the morphology of the population was determined by the number of positive correlations with significant differences (*p* < 0.05), including a correlation test using Spearman’s method. In order to reduce the matrix of variables, a principal components analysis was performed, and it was evaluated based on Kaiser’s criteria (eigenvalue > 1). Finally, a hierarchical analysis of conglomerates using Ward’s method was performed using the Euclidean distance to evaluate the distances among localities. Morphometric variables were also included to visualize the relationship among the localities and their average per variable. The results showed that the animals evaluated presented a certain degree of homogeneity and maintained a highly harmonic model. The BCG population showed a high aptitude for milk production, which confirmed the zootechnical purpose of the breed. The BCG populations evaluated maintain similar morphostructural profiles specific to them that can distinguish this population from other animal breeds.

## 1. Introduction

Currently, the different species of livestock raised have different production purposes to contribute to the family economy. For this reason, many of the most vulnerable people often maintain a diversity of species in their production systems [1]. With regard to the aforementioned benefits, the goat is no exception. Since its domestication, it has provided man with meat, milk, and skin, coupled with its value for its adaptability and resilience [2]. With great easiness, goats adapt to extreme temperatures, undernourishment, high altitudes, long walking distances, and long periods of drought [3,4].

Mexico has more than 8.7 million goats [5], most of which are kept in semi-extensive systems with minimal resources and represent a multifunctional asset that has a great impact on the livelihood of people living in marginalized communities [6,7].

In addition, the importance that these goats have in local populations lies in their role as reservoirs for genetic diversity given the future challenges that humanity could face, for example, climate change, increasing world population, and increasing demand for food, among others [8]. A representative sample of these local populations as evidence of zoogenetic resources in Mexico is the Black Creole goat (BCG) that has endured for more than 500 years in the traditional production systems of the central region [9]. Currently, the importance of the BCG is based on the quality of its milk, which compared to other breeds (Alpine and Nubian), has higher solids contents and, therefore, a higher cheese yield, which translates into greater economic gains for the producer. In addition to this benefit, it is also important to emphasize the great capacity of adaptation and resistance that the BCG has developed in environments with scarce forage, such as the semi-desert of central Mexico [10]. However, in the same way as other Creole populations in the rest of the world, their existence is threatened by the importation of animals of specialized breeds.

A decade ago, it was reported that traditional production systems require multipurpose animals that, while appearing less productive, may contain valuable functional traits [11]. As a result, interest has emerged in characterizing and documenting the particularities of local animals, with the aim of establishing proper management practices and the conservation of diversity [7]. A typical tool in the description of local populations is zoometry, a tool that allows knowing the productive capacities of domestic ruminants or their inclination towards a certain productive aptitude, through the interpretation of functional indices for each individual [12,13]. Similarly, the analysis of the principal components of zoometric measurements provides useful information for racial diagnosis, the determination of somatic states, or to determine the sexual dimorphism of a race, among others [14].

The objective of this study is the morphostructural characterization of the BCG, providing an objective description of the body shape and structure of this population.

## 2. Materials and Methods 

This project was approved by the Bioethics Committee of the Natural Sciences Department of the Autonomous University of Queretaro under registry number 22FCN2016. The measurements were made considering animal welfare and health, according to the Mexican Official Norm NOM-051-ZOO-1995 and Mexican Federal Law DOF 25-07-2007.

### 2.1. Sampling

**Figure 1:**
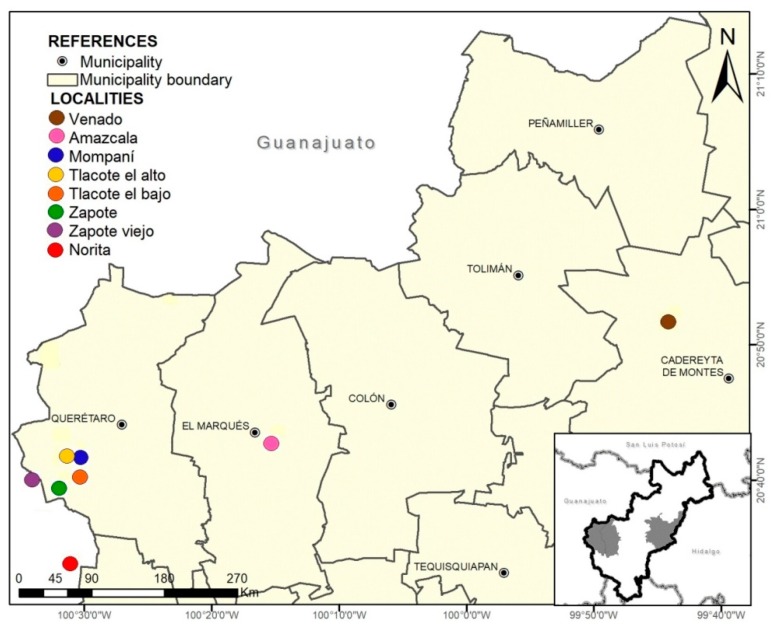
Localities where the herds included in the study were located.

The study was carried out with 226 animals (216 females and 10 males) that, per dentition, were proved to be older than one year of age. The animals were distributed across eight localities in central Mexico: Norita (*n* = 20) and Zapote viejo (*n* = 38), belonging to the state of Guanajuato; Venado (*n* = 11), Amazcala (*n* = 15), Tlacote el alto (*n* = 22), Tlacote el bajo (*n* = 38), Zapote (*n* = 59) and Mompaní (*n* = 23), belonging to the state of Querétaro (Figure 1).

The sample size (*n*) was determined using the following equation:

(1) $$n\;=\;{\left(\frac{z}{m}\right)}^{2}p\left(1-p\right)$$

where: *z* is the value of *z* obtained from the cumulative probability values table for the standard normal distribution (1.64 for a 90% confidence level); *m* is the margin of error (0.05 = ±5%), and *p* is the estimated value of the proportion of the sample that will respond to the survey in the same sense, for this case, it was considered 0.3 [15]. The choice of herds included in the study was made by the non-probabilistic snowball method, where each herd owner was asked for a reference to others that met the target population, having these references suggest the following members of the sample, and so on until completing the total sample [16].

### 2.2. Morphometric Variables

**Figure 2:**
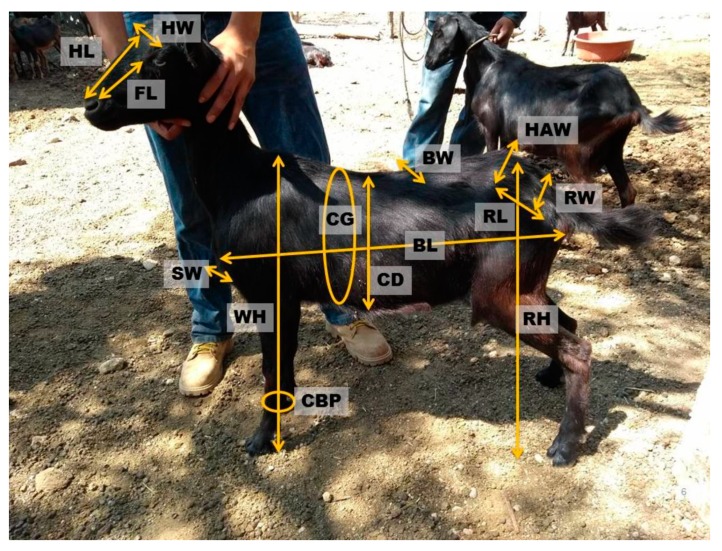
Morphometric variables analyzed: head width (HW), head length (HL), face length (FL), wither height (WH), chest girth (CG), body length (BL), bicostal width (BW), chest depth (CD), shoulder width (SW), rump length (RL), rump width (RW), haunch width (HW), rump height (RH), and cannon bone perimeter (CBP).

The variables analyzed were: head width (HW), which indicates the distance between the most lateral points of the zygomatic arches; head length (HL), the distance from the occipital protuberance to the upper lip; face length (FL), which indicates the measurement of the midpoint of the zygomatic arches to the upper lip; wither height (WH), which is the distance from the ground to the highest point of the cross region; chest girth (CG), which measures the contour of the thorax, passing through the subesternal hollow and the process of the 7th–8th dorsal vertebrae; body length (BL), from the region of the stop to the tip of the buttock; bicostal width (BW), which indicates the distance from one side to the other at the elbows; chest depth (CD), from the lowest point of the wither region to the sternum; shoulder width (SW), the measurement of the thickness of the chest at the front of the animal; rump length (RL), from the tip of the haunch to the tip of the buttock; rump width (RW), indicating the distance among the two external iliac tuberosities; haunch width (HW), measures the distance between the hip joints; rump height (RH), from the ground to the highest point of the rump; cannon bone perimeter (CBP), measures the circumference of the middle third of the metacarpal bone [14,17] (Figure 2). Measurements were taken using a caliper (Haglöf Mantax Blue^®^) as a zoometric cane and a flexible tape measure following the Food and Agriculture Organization of the United Nations (FAO) methodology [18].

Four indices of ethnological interest were also included: corporal index (*COI = BL × 100/CG*); thoracic index (*THI = BW × 100/CD*); cephalic index (*CEI = HW × 100/HL*); pelvic index (*PEI = RW × 100/RL*). Five indices of productive interest were also calculated: proportionality index (*PRI = WH × 100/BL*); metacarpal–thoracic index (*MTI = CBP × 100/CG*); relative depth of thorax index (*RDI = CD × 100/WH*); transversal pelvic index (*TPI = RW × 100/WH*); longitudinal pelvic index (*LPI = RL × 100/WH*) [14,17].

### 2.3. Statistical Analysis

The descriptive statistics (mean, standard deviation, coefficient of variation, maximums and minimums) were calculated for the morphometric variables and the nine zoometric indices included in the study.

The morphological harmony of the BCG was determined by the number of positive correlations with significant differences (*p* < 0.05), including a correlation test by the Spearman method because the data did not follow a normal distribution (Kolmogorov–Smirnov *p* < 0.05 normal test). A highly harmonic model is where the number of correlations exceeds 50%, a medium harmonic model is when they are close to 50%, and a low harmonic model is when 25% of the correlations are significant and positive [19].

A principal components analysis (PCA) was performed to reduce the matrix of morphometric variables to a small number of non-correlated variables called components. The main components obtained were evaluated based on Kaiser’s criterion (eigenvalue > 1) [20].

Finally, a hierarchical analysis of conglomerates using Ward’s method was carried out using the Euclidean distance to construct a dendrogram and to evaluate the distances among localities. Morphometric variables were also included in this analysis and a heatmap was generated to visualize the relationship among the localities and their average per variable. All statistical analyses were performed using the statistical programming language R [21].

## 3. Results

The descriptive statistics for the fourteen morphometric variables are shown in Table 1. The obtained results show that the evaluated animals are homogeneous in most of the variables when the coefficients of variation (CV) were lower than 10% in ten of the fourteen variables studied; this indicates that the average values for each variable were significant enough to consider the estimates as acceptable. Some authors argue that when the CV exceeds 30%, the precision is too low, thus the data should be discarded [22,23]. Data were not discarded because the highest CV was 23.32%, obtained for the bicostal width (BW). However, the measurements of each individual can be influenced by several factors, such as those related to the production system: the availability of fodder, development, age, number of births, and nutritional status, among others [24].

The correlation analysis confirmed a highly harmonic model for the study population, presenting 91.21% of the positive and significant correlations. The highest correlation estimations found were between HL and FL, followed by WH and RH, with values of 0.87 and 0.78, respectively (Table 2).

With respect to the zoometric indices, the THI had the highest coefficient of variation (19.26%) due to the fact that this index included the BW variable, which was the one with the greatest variation in the present study. The rest of the zoometric indices calculated were more homogeneous, with a coefficient of variation ranging from 7.63 to 19.26 for RDI and THI (Table 3).

The PCA considered the first three principal components (PC) to be significant under Kaiser’s criteria, with eigenvalues greater than 1 and a communality value ≥ 0.5. The first PC represented 45.64% of the observed variance (eigenvalue 6.39) and describes mostly the trunk and extremities for obtaining the highest values for CD, SW, CBP, and CG; however, for this PC, all the variables had a high load except BW and RL, with values less than 0.5. As indicated by the PCA, variables that could not be explained by the first component could be taken up by the second component and so on successively. For PC2, the variable that had more contribution was RL (0.77), and for PC3, the variables corresponded to the description of the head’s structure (HL and FL) (Table 4). 

**Figure 3:**
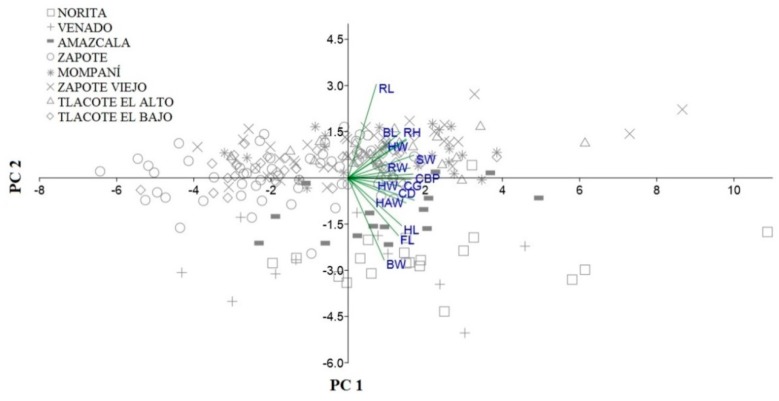
Principal components analysis of fourteen morphometric variables in the Black Creole goat from eight localities in central Mexico. (HW) head width, (HL) head length, (FL) face length, (WH) wither height, (CG) chest girth, (BL) body length, (BW) bicostal width, (CD) chest depth, (SW) shoulder width, (RL) rump length, (RW) rump width, (HAW) haunch width, (RH) rump height, (CBP) cannon bone perimeter.

Together, PC1, PC2, and PC3 represented 67.49% of the total variance and the values obtained indicated that the anterior part of the body of the animals was described by PC3, the middle part by PC1, and the posterior part was described by PC2. The values of PC1 and PC2 of the animals per locality are represented graphically in Figure 3. The animals of the eight localities were homogeneous, so they were scattered without generating groups or subpopulations.

**Figure 4:**
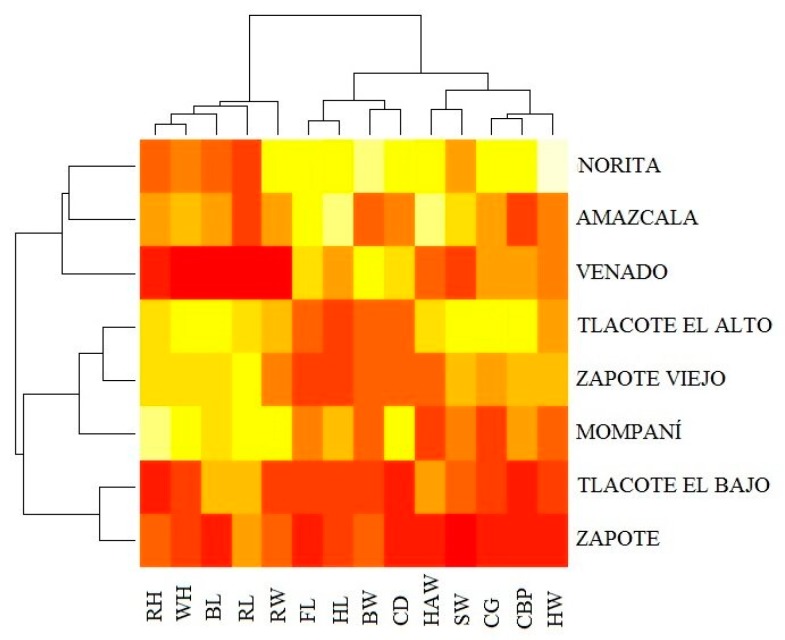
Dendrogram by localities elaborated using Ward’s method and Euclidean distances. (HW) head width, (HL) head length, (FL) face length, (WH) wither height, (CG) chest girth, (BL) body length, (BW) bicostal width, (CD) chest depth, (SW) shoulder width, (RL) rump length, (RW) rump width, (HAW) haunch width, (RH) rump height, (CBP) cannon bone perimeter.

The hierarchical analysis of conglomerates generated a dendrogram that clustered the localities included in the study into three groups that coincided with the place of origin of the animals (Figure 4). The first group included Norita, Amazcala, and Venado. The second group, formed by Mompaní, Zapote Viejo, and Tlacote el Alto, showed the similarity that exists among these populations. This is in agreement with the nature of the data, these herds were originated by mixtures of populations from the third group, which includes populations from Zapote and Tlacote el Bajo, which were pioneers in the breeding of the BCG. The distances among localities are shown in Table 5. The smallest distance obtained was between the localities of Zapote Viejo and Tlacote el Alto (2.34), while the greatest distance was generated between Zapote and Norita (7.77).

## 4. Discussion

The results obtained show that the use of morphometric variables represents a first approximation in the characterization of populations and sets precedent for the management of animal genetic resources, even though some authors have considered that they are more sensitive to environmental and human factors [25].

Of the fourteen variables included in the study, ten (HW, HL, WH, CG, BL, CD, SW, RL, RW, CBP) had been considered in the morphostructural evaluation of Spanish goat breeds. The BCG obtained values similar to those reported for the Granadina and Malagueña breeds, with the exception of the HL, RL, and SW variables, where these breeds presented higher values. This gives us an indication of the origin of the BCG and its morphological similarity with the Granadina breed, maintaining morphological characteristics of Spanish dairy goats [26]. With respect to local goats from Mexico, this type of study has been carried out mostly with goats from the Mixteca region. In the present study, the populations of BCGs obtained values similar to those reported for the Creole goats of the state of Puebla, Mexico, with respect to the variables HW, HL, WH, RH, and CD; however, it is noted that the BCG presents a larger size for CG, RW, and RL, which shows that this is an animal with greater thoracic capacity and greater development of the rump [27].

The number of significant positive correlations among body measurements (91.21%) showed a high degree of morphological harmony in the BCG. This model is based on the fact that the increase or decrease in one of the variables will mean an increase or decrease for another; however, the relation among variables must be kept in proportion to the first one in order for the fundamental structure to be maintained too [19].

Taking into account these results and the CV obtained, it can be stated that the BCG is an animal adapted to the conditions of the environment where it develops, in this way it maintains its homogeneous morphostructure.

With respect to the zoometric indices, the COI shows the proportionality of the breed and allows to classify the animals according to baronian systematics in brevilinear (≤85), mesolinear (>86 and <88) or longilinear (≥90). The value obtained in the present study (85.11) indicates that the BCG has a brevilinear profile, with transversal measures predominating over length measures. The THI shows the variations in the shape of the thorax, which is greater or circular in meat cattle (≥89), and smaller or elliptical in dairy cattle (≤85). The THI obtained for the BCG suggests that it is a dairy type animal [14]. Similar values were obtained for the breeds Blanca of Rasquera and Colorada Pampeana, which show a short and wide animal with an elliptical thorax [28].

Because of the somation of cephalic variables (HW and HL) included for the CEI which are not influenced by environmental or management factors, this index is important for the racial characterization of the animals. The CEI measures the proportionality of the head and classifies it as brachycephalic (CEI > 100), mesocephalic (CEI = 100), and dolichocephalic (CEI < 100), resulting in dolichocephalous for the BCG (74.29), which is similar to that reported for the Majorera and Tinerifeña breeds, as well as the semi-wild goat [29].

The PEI indicates that the ratio of pelvic width to pelvic length is related to reproductive fitness. The result shows convexilinear animals (PEI < 100), where the rump length predominates over the width [14].

Proportionality index (PRI) has a special focus on the shape of animals, indicating that at a lower value, the shape of the animal resembles a rectangle—the predominant shape in meat-suitable animals. The PRI in the BCG showed low meat suitability, represented in square form, characteristic of dairy cattle, higher than the Creole goat of the central valleys of Oaxaca, Mexico [30].

Metacarpal–thoracic index (MTI) establishes the relationship between the mass of the animal and the members that support it, classifying animals as hypermeter (>11), eumeter (>10 and <11) or ellipometric (<10), with higher value in meat breeds. The value obtained in this study (10.14) indicates that the population has a eumometric tendency, similar to the Colorada Pampeana goat of Argentina [28].

The RDI shows the relationship between chest depth and leg length, with values greater than 50 in short-legged animals with a tendency to a meat phenotype. The BCG has low meat suitability and is mostly oriented towards milk production.

As regards to the TPI and LPI, these are an estimate for the meat suitability of the breed. A TPI greater than 33 and an LPI less than 37 are indicators of meat breeds. The BCG population showed low values for the TPI (22.30) and LPI (26.87), which means that it has a medium tendency that gives it the opportunity to develop muscle tissue as well. These last three indices are similar to those reported for the Creole goat of the central valleys of Oaxaca, Mexico [30].

With respect to the PCA, three components were significant and explain 67.50% of the total variance. A similar percentage (67.80%) of explanation of the total variance was also obtained in local goats from the south of the state of Mexico, taking into account the first three components and ten variables [31]. The same percentage (67.82%) was obtained for the Brazilian breed Canindé, using four components and eleven morphometric variables, and like the BCG, only three components had eigenvalues greater than one [32]. Goats from northern Morocco with 16 variables also obtained similar values for the percentage of variance, with three components explaining 66.96% of the variance [33]. In Indian goats, using 11 morphometric variables, four components were significant, which explained 85.84% of the total variance [34]. In another work with local goats, using only four morphometric variables, it was found that the first two components explained a large percentage of the total variance (94.15% in females and 97.65% in males); however, only the first component was significant [35]. Although the number of variables was not so small, the first components can explain more than 90% of the total variance [36].

Head length (HL) and face length (FL) were the variables of greater contribution for the three components, because their variance is better explained by the components (87% and 84%, respectively). The contribution of cephalic variables has been described in other studies, demonstrating that these characteristics are important to describe the race and define the cephalic profile of the animals [32].

The influence of each variable can change from one population to another and it is difficult to compare among populations. This is the case for the CBP, which for the Canindé breed is the variable that has the least contribution in the model (18.5%) [32], compared with the BCG (66%) and local goats in India (87%) [34]. As reported by other authors, the variation shown is the result of the absence of selection or it is also produced because different parts of the body are more affected by the environment than by other factors. However, head length is important in several studies due to its close relationship with the cranial bone, which is not strongly influenced by other factors [12].

Hierarchical cluster analysis demonstrates the relationships between morphometric variables and the localities included in the study. The RH, WH, BL, RL, and RW variables that help to describe the height and length of the animals had the greatest effect in grouping the localities of Tlacote el Alto, Zapote viejo, and Mompaní in a single cluster. The relationship among these variables (RH, WH, and BL) has also been observed for Canindé goats [32]. The cluster formed by the localities of Norita, Amazcala, and Venado was influenced by having higher values for the variables FL, HL, BW, and CD, which are shown in lighter colors in the heatmap (Figure 4). A third group (Tlacote el bajo and Zapote) comprises the lowest measurements for most of the variables, mainly for CG, CBP, and HW (more intense colors in the heatmap). Other works show the effects of the variables for classification by locality; however, it is emphasized that the genetic relationship cannot be easily derived from phenotypic similarity because genotypes can be mostly related to productive traits [25]. Since it has been proven that environmental factors in different production systems have an effect on the morphostructure of animals, it would be useful to include these elements in further characterization studies [37].

## 5. Conclusions

The evaluated herds of Black Creole goat represent a morphologically homogeneous population, preserving a high harmonic model. This indicates that it is a population adapted to the conditions of the environment and the production system, taking into account that these factors, environment and management, mainly influence the morphostructural development of the animals. Based on the morphostructure, the Black Creole goat is dolichocephalic, with an elliptical thorax characteristic of dairy animals. The indices of productive interest also indicate in their majority an animal oriented to milk production; however, the potential of these animals is still unknown, a reason for why the production of meat cannot totally be discarded.

Finally, the present study could be the basis for future decisions on Black Creole goat’s management, improvement, and conservation, considering that a genetic analysis would complement the characterization of the breed.

## Figures and Tables

**Table 1:** Descriptive statistics of the morphometric variables used in Black Creole goats older than one year of age (*n* = 226).

Variable	Mean	SD	CV	Min	Max
HW	11.56	0.98	8.50	9.50	18.00
HL	15.66	1.40	8.96	11.00	20.50
FL	12.74	1.45	11.38	9.00	19.50
WH	70.12	4.31	6.14	57.00	88.00
CG	84.07	7.36	8.76	56.00	106.00
BL	71.20	6.11	8.59	52.00	87.00
BW	21.88	5.10	23.32	15.60	41.00
CD	30.31	2.63	8.69	24.00	38.00
SW	16.86	1.53	9.07	13.00	24.00
RL	18.81	2.04	10.85	13.00	27.00
RW	15.61	1.40	8.95	12.50	21.00
HAW	17.17	1.79	10.43	12.20	23.00
RH	70.54	3.77	5.34	61.00	86.00
CBP	8.49	0.68	8.06	6.20	11.00

SD = standard deviation, CV = coefficient of variation, Min = minimum value, Max = maximum value. (HW) head width, (HL) head length, (FL) face length, (WH) wither height, (CG) chest girth, (BL) body length, (BW) bicostal width, (CD) chest depth, (SW) shoulder width, (RL) rump length, (RW) rump width, (HAW) haunch width, (RH) rump height, (CBP) cannon bone perimeter.

**Table 2:** Spearman correlation matrix among morphological variables of the Black Creole goat.

	HL	BW	HAW	CD	HW	CG	CBP	RW	SW	RH	WH	BL	RL
**FL**	0.87 ***	0.24 ***	0.41 ***	0.49 ***	0.28 ***	0.28 ***	0.36 ***	0.26 ***	0.32 ***	0.35 ***	0.31 ***	0.23 ***	0
	**HL**	0.25 ***	0.39 ***	0.52 ***	0.27 ***	0.31 ***	0.33 ***	0.38 ***	0.34 ***	0.38 ***	0.34 ***	0.24 ***	0.03
		**BW**	0.43 ***	0.51 ***	0.35 ***	0.57 ***	0.38 ***	0.4 ***	0.32 **	0.15	0.17	0.2	−0.11 ***
			**HAW**	0.46 ***	0.39 ***	0.59 ***	0.45 ***	0.58 ***	0.58 ***	0.32 ***	0.34 ***	0.42 ***	0.14
				**CD**	0.48 ***	0.65 ***	0.59 ***	0.57 ***	0.53 ***	0.49 ***	0.52 ***	0.48 ***	0.22 *
					**HW**	0.43 ***	0.57 ***	0.44 ***	0.42 ***	0.32 ***	0.43 ***	0.35 ***	0.18
						**CG**	0.55 ***	0.58 ***	0.64 ***	0.44 ***	0.44 ***	0.49 ***	0.32 ***
							**CBP**	0.5 ***	0.56 ***	0.42 ***	0.47 ***	0.49 ***	0.32 ***
								**RW**	0.59 ***	0.49 ***	0.44 ***	0.48 ***	0.4 ***
									**SW**	0.48 ***	0.46 ***	0.55 ***	0.4 ***
										**RH**	0.78 ***	0.43 ***	0.44 ***
											**WH**	0.49 ***	0.4 ***
												**BL**	0.48 ***

(HW) head width, (HL) head length, (FL) face length, (WH) wither height, (CG) chest girth, (BL) body length, (BW) bicostal width, (CD) chest depth, (SW) shoulder width, (RL) rump length, (RW) rump width, (HAW) haunch width, (RH) rump height, (CBP) cannon bone perimeter. * *p* < 0.05, ** *p* < 0.01, *** *p* < 0.001.

**Table 3:** Descriptive statistics of zoometric indices.

INDEX	MEAN	SD	CV	Min	Max
COI	85.11	8.72	10.24	62.07	151.79
THI	71.98	13.87	19.26	50.00	117.14
CEI	74.29	9.03	12.16	57.89	163.64
PEI	83.75	10.24	12.22	59.26	121.43
PRI	98.98	8.10	8.18	75.00	142.31
MTI	10.14	0.84	8.29	7.75	14.52
RDI	43.27	3.30	7.63	34.78	55.07
TPI	22.30	1.89	8.46	17.95	29.17
LPI	26.87	2.80	10.42	19.57	39.13

SD = standard deviation, CV = coefficient of variation, Min = minimum value, Max = maximum value. COI = corporal index, THI = thoracic index, CEI = cephalic index, PEI = pelvic index, PRI = proportionality index, MTI = metacarpal–thoracic index, RDI = relative depth of thorax index, TPI = transversal pelvic index, LPI = longitudinal pelvic index.

**Table 4:** Eigenvalues, proportion of total variance, and correlations between variables and principal components (PCs) in the Black Creole goat.

Variables	PC 1	PC 2	PC 3	Communality
HW	0.63	−0.07	−0.32	0.51
HL	0.65	−0.39	0.55	0.87
FL	0.61	−0.47	0.50	0.84
WH	0.70	0.31	0.25	0.66
CG	0.76	−0.01	−0.29	0.66
BL	0.63	0.37	−0.08	0.54
BW	0.44	−0.67	−0.41	0.81
CD	0.80	−0.18	0.00	0.67
SW	0.80	0.19	−0.14	0.69
RL	0.34	0.77	0.02	0.71
RW	0.75	0.08	−0.12	0.59
HAW	0.70	−0.20	−0.10	0.54
RH	0.70	0.32	0.34	0.71
CBP	0.78	0.03	−0.21	0.66
Eigenvalue	6.39	1.88	1.18	
% Variance	45.64	13.41	8.45	

(HW) head width, (HL) head length, (FL) face length, (WH) wither height, (CG) chest girth, (BL) body length, (BW) bicostal width, (CD) chest depth, (SW) shoulder width, (RL) rump length, (RW) rump width, (HAW) haunch width, (RH) rump height, (CBP) cannon bone perimeter.

**Table 5:** Matrix of Euclidean distances among studied localities.

	Norita	Venado	Amazcala	Zapote	Mompaní	Z. Viejo	T. Alto
Venado	5.11	-					
Amazcala	4.81	5.23	-				
Zapote	7.77	4.91	6.07	-			
Mompaní	6.04	6.60	4.71	5.70	-		
Z. Viejo	5.88	5.81	4.77	4.79	3.25	-	
T. Alto	5.55	6.64	4.50	6.43	4.03	2.34	-
T. Bajo	7.03	4.92	4.82	2.60	5.17	3.68	4.97
